# Retinal capillary rarefaction is associated with arterial and kidney damage in hypertension

**DOI:** 10.1038/s41598-020-79594-3

**Published:** 2021-01-13

**Authors:** Shaun Frost, Janis Marc Nolde, Justine Chan, Anu Joyson, Cynthia Gregory, Revathy Carnagarin, Lakshini Y. Herat, Vance B. Matthews, Liam Robinson, Janardhan Vignarajan, David Prentice, Yogesan Kanagasingam, Markus P. Schlaich

**Affiliations:** 1 Commonwealth Scientific and Industrial Research Organisation (CSIRO) Health and Biosecurity, Brockway Rd Floreat 6014, Private Bag 5, Wembley, WA 6913 Australia; 2 Australian E-Health Research Centre, Perth, Australia; 3 Perron Institute for Neurological and Translational Science, Perth, Australia; 4 Dobney Hypertension Centre, School of Medicine - Royal Perth Hospital Unit/Medical Research Foundation, University of Western Australia, Perth, Australia; 5 Departments of Cardiology and Nephrology, Royal Perth Hospital, Perth, Australia; 6 Neurovascular Hypertension and Kidney Disease Laboratory, Baker Heart and Diabetes Institute, Melbourne, Australia; 7 School of Medicine, University of Notre Dame Australia, Fremantle, Australia

**Keywords:** Diagnostic markers, Kidney, Hypertension, Cardiovascular biology

## Abstract

Microvascular disease and rarefaction are key pathological hallmarks of hypertension. The retina uniquely allows direct, non-invasive investigation of the microvasculature. Recently developed optical coherence tomography angiography now allows investigation of the fine retinal capillaries, which may provide a superior marker of overall vascular damage. This was a prospective cross-sectional study to collect retinal capillary density data on 300 normal eyes from 150 hypertensive adults, and to investigate possible associations with other organ damage markers. The average age of participants was 54 years and there was a greater proportion of males (85; 57%) than females. Multivariate, confounder adjusted linear regression showed that retinal capillary rarefaction in the parafovea was associated with increased pulse wave velocity (*β* = − 0.4, *P* = 0.04), log-albumin/creatinine ratio (*β* = − 0.71, *P* = 0.003), and with reduced estimated glomerular filtration rate (*β* = 0.04, *P* = 0.02). Comparable significant associations were also found for whole-image vascular-density, for foveal vascular-density significant associations were found with pulse wave velocity and estimated glomerular filtration rate only. Our results indicate that retinal capillary rarefaction is associated with arterial stiffness and impaired kidney function. Retinal capillary rarefaction may represent a useful and simple test to assess the integrated burden of hypertension on the microvasculature irrespective of current blood pressure levels.

## Introduction

Hypertension has been identified as one of the most significant risk factors for global morbidity and mortality^1^. It significantly increases the risks of stroke, coronary heart disease, heart failure and kidney disease and is a major cause of premature death worldwide. Microvascular disease and rarefaction are key pathological hallmarks of hypertension^2^. Subtle damage to organs such as the heart, kidney, eye and the peripheral vasculature can be detected early in the disease process and may help to prevent clinical events. Diagnostic assessment of hypertension mediated organ damage (HMOD) allows better prediction of cardiovascular (CV) risk^3–5^, identifying high-risk individuals for whom a more intense treatment is needed. Successful treatment can provide significant reductions in risk of subsequent CV disease^6,7^.

The retina uniquely allows direct, non-invasive investigation of the microvasculature, providing a valuable window into the microvascular sequelae of hypertension. Assessment of retinal damage due to hypertension (hypertensive retinopathy) is recommended in clinical guidelines for the management of patients with hypertension^8^. Hypertensive retinopathy progresses through various stages beginning with general arteriolar narrowing and subsequently focal areas of arteriolar narrowing, opacification of arteriolar walls, arteriovenous nipping, hemorrhages, hard exudates and cotton-wool spots. Signs of hypertensive retinopathy are predictive of incident stroke, congestive heart failure, and CV mortality—independent of traditional risk factors^9–16^. Even the earliest stage of hypertensive retinopathy- general arterial narrowing, is associated with stroke^13,17,18^, kidney disease^19,20^ and CV mortality^21,22^. Digital fundus photography can detect these retinal changes, but cannot resolve the finer retinal capillaries, which are more representative of the entire microvascular network and may provide the earliest markers of ischemia/hypoxia, before arteriolar and venular changes occur. Early detection of hypertensive damage is important to prevent further degeneration and recent evidence suggests that retinal neurodegeneration is already evident in individuals with mildly elevated blood pressure (BP) levels^23^.

**Figure 1:**
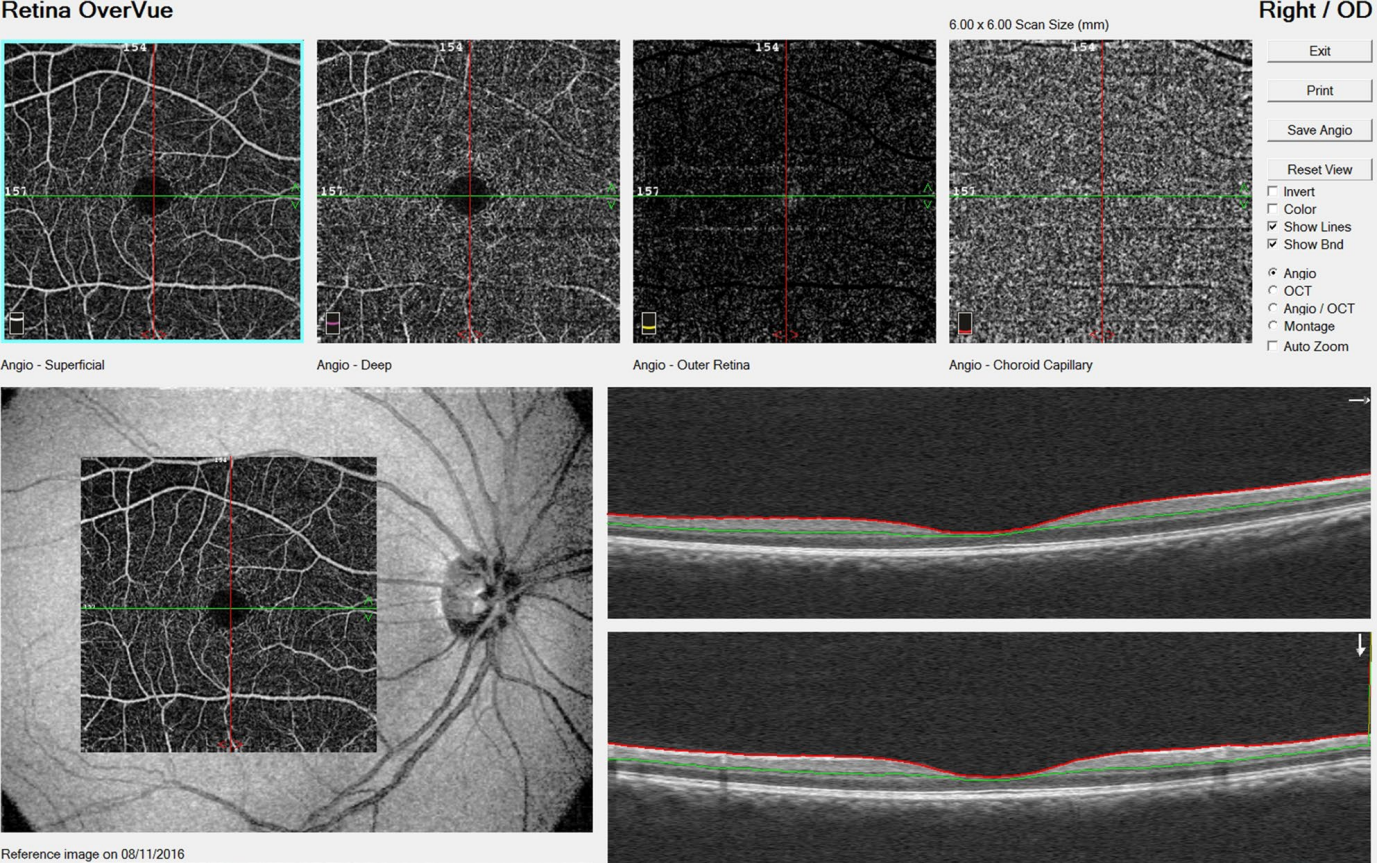
OCT angiogram from a single eye, showing the four retinal depths (top): the superficial retinal plexus, the deep retinal plexus, the outer retina and the choriocapillaris. Bottom left shows the scan region (darker) overlaid on the broader retina. Bottom right shows the cross-section of the superior vascular plexus (SVP).

Optical coherence tomographic angiography (OCT-A) is a more recent ocular imaging technology that permits fast, non-invasive assessment of the retinal capillaries and blood flow in the retina (see Fig. 1). Importantly, OCT-A can be used to visualize all layers of the retinal vasculature in detail. Recent investigations using OCT-A technology have reported reduced retinal capillary density in diabetes^24^ and uncontrolled hypertension^25^, while another study reported restoration of normal capillary density after treatment with carotid angioplasty and stenting^26^.

This study aimed to further investigate retinal capillary changes with respect to BP and HMOD measures related to vascular stiffness and kidney function. Kidney function was assessed using urinary albumin/creatinine ratio (ACR) and estimated glomerular filtration rate (eGFR), and arterial stiffness was assessed using carotid-femoral pulse wave velocity (PWV).

## Results

The clinical characteristics of the study population are shown in Table 1. Overall 150 patients were included in the analysis. The mean ± SD age of participants was 54 ± 16 years and there was a greater proportion of males (n = 85, 57%) than females (n = 65, 43%). Seventy nine percent of patients received treatment with some form of antihypertensive medication. Of those taking hypertensive medication, the average number of antihypertensive drugs was 1.8 ± 0.7. Thirty-two participants were on no antihypertensive medication (usually due to treatment with lifestyle interventions or intolerances to anti-hypertensive medication), 43 on only one medication, 55 on a combination of two medications and 20 on triple anti-hypertensive therapy. Further baseline characteristics of the included patients are described in Table 1. Data on body mass index (BMI) was missing in 5% of the cases, medical history (type 1 or 2 diabetes) in 8%, PWV in 15%, eGFR in 16%, ACR in 26% of cases. All other data described in Table 1 was complete.

**Table 1:** Continuous clinical characteristics of the analyzed patient cohort.

Parameter	All participants (*n* = 150)	eGFR < 60 ml/min/1.73 m^2^ (n = 19)	eGFR ≥ 60 ml/min/1.73 m^2^ (n = 106)
Age (years)	53.8 (15.7)	64.8 (12.4)	53.0 (15.2)
Sex (male/female)	85/65	13/6	62/44
BMI (kg/m^2^)	32.1 (6.8)	33.8 (7.4)	31.8 (6.7)
Ambulatory systolic blood pressure (mmHg)	134.9 (17.7)	136.7 (18.5)	135.8 (16.9)
Ambulatory diastolic blood pressure (mmHg)	81.3 (12.3)	72.1 (12.9)	82.4 (10.9)
Foveal density (%)	34.0 (7.2)	30.9 (6.8)	35.3 (6.9)
Para foveal density (%)	52.5 (4.4)	49.5 (4.9)	52.8 (4.2)
Whole image density (%)	49.6 (3.9)	46.8 (4.0)	49.9 (3.7)
PWV (m/s)	8.8 (2.1)	10.0 (1.6)	8.7 (2.0)
eGFR (ml/min/1.73 m^2^)	78.0 (19.9)	–	–
≥ 60/< 60	106/19	–	–
ACR (%)	8.0 (27.2)	43.0 (67.4)	2.6 (4.1)
HbA1c (%)	6.0 (1.2)	6.3 (1.2)	6.0 (1.2)

Data are given as mean (SD). BMI, body mass index; blood pressure data from ambulatory 24 h analysis; PWV, carotid-femoral pulse wave velocity; eGFR, estimated glomerular filtration rate; ACR, albumin-to-creatinine ratio; HbA1c, glycated hemoglobin.

Of the entire cohort, 46 had T2D and 1 had type 1 diabetes. Data on HbA1c was available for 76% of the cohort and was utilised to categorise a further n = 2 participants (with missing diabetes history) as having diabetes (based on HbA1c > 6.5%)^27^, resulting in 48 participants with diabetes in the cohort, and data on diabetes unavailable for 7% of the full cohort.

The cohort had an average BMI of 32.1 kg/m^2^ which placed them into the obese range. The average 24 h ambulatory BP was 135/81 mmHg.

**Figure 2:**
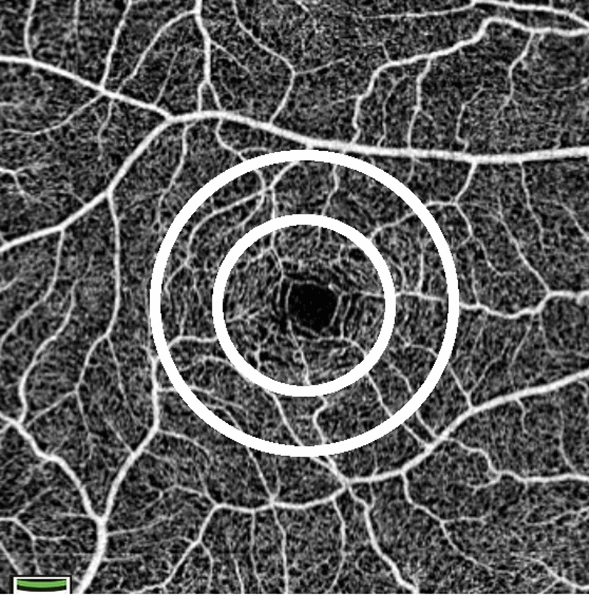
OCT angiogram of the left macula illustrating the regions of interest for retinal capillary density analysis. The central circular fovea region has a diameter of 1.5 mm, the parafovea ring reaches from 1.5 to 2.5 mm from the foveal center and the whole image is a 6 mm square centered on the fovea.

**Figure 3:**
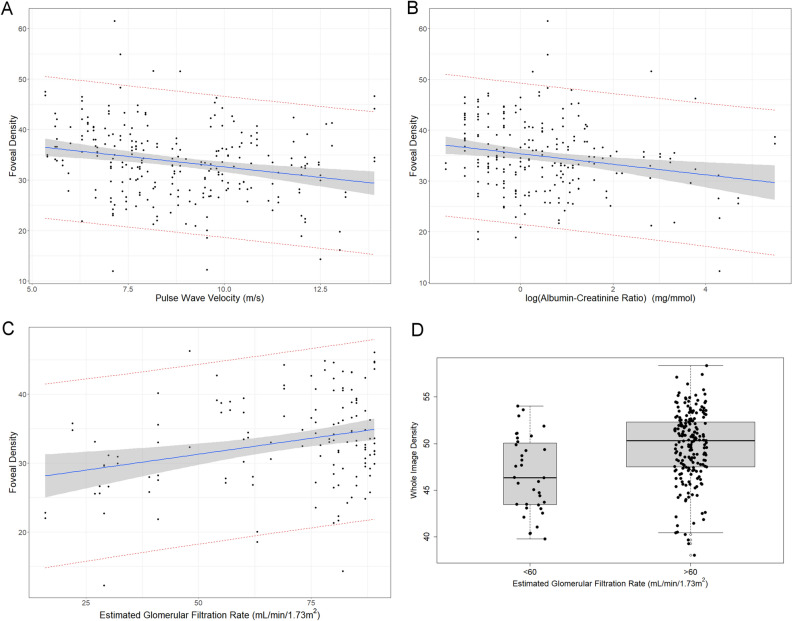
Scatterplots of foveal density against measures of hypertension mediated organ damage, including the unadjusted linear regression model fits (blue line) with the mean (grey area) and observed (red dashed lines) 95% confidence intervals. (**a**) Pulse wave velocity; (**b**) log (albumin–creatinine ratio); (**c**) estimated glomerular filtration rate (eGFR > 90 excluded), (**d**) boxplot comparing whole image density between groups classified by estimated glomerular filtration rate <  > 60).

Capillary density was calculated for 3 zones of interest, each centered on the fovea (see Fig. 2). The association between retinal capillary density at SVP and systemic factors is shown in Table 2 and Fig. 3. Univariate linear regression showed that retinal capillary rarefaction in each zone of interest was associated with increased PWV (*P* < 0.0001 to *P* = 0.006) and logACR (*P* = 0.0001 to *P* = 0.02), and with reduced eGFR (*P* < 0.0001 to* P* = 0.005). These results remained statistically significant after multiple testing adjustment.

**Table 2:** Univariate model results.

	*N (eyes)*	Foveal density			Para foveal density			Whole image density		
		Coefficient	95% CI	*P* value	Coefficient	95% CI	*P* value	Coefficient	95% CI	*P* value
BP control status										
Well controlled BP (ref)										
Poorly controlled BP	300	0.20	− 1.98 to 2.38	0.86	− 0.31	− 1.51 to 0.89	0.6	− 0.37	− 1.45 to 0.72	0.5
Ambulatory BP (mmHg)										
SBP	300	0.002	− 0.05 to 0.06	0.9	− 0.03	− 0.06 to 0.01	0.1	− 0.02	− 0.04 to 0.01	0.2
DBP		0.01	− 0.07 to 0.09	0.7	0.06	0.01 to 0.11	**0.01**	0.05	0.01 to 0.09	**0.01**
PWV (m/s)	254	− 0.83	− 1.42 to − 0.24	**0.006**	− 0.74	− 1.02 to − 0.46	**< 0.0001**	− 0.76	− 1.03 to − 0.49	**< 0.0001**
eGFR (mL/min/1.73 m^2^)	250	0.10	0.05 to 0.15	**< 0.0001**	0.07	0.03 to 0.10	**0.0001**	0.07	0.04 to 0.09	**< 0.0001**
eGFR (mL/min/1.73 m^2^) (eGFR > 90 excluded)	136	0.09	0.03 to 0.16	**0.005**	0.07	0.03 to 0.12	**0.001**	0.07	0.04 to 0.10	**< 0.0001**
eGFR (< 60 mL/min/1.73 m^2^) (reference eGFR ≥ 60)	250	4.21	1.12 to 7.31	**0.007**	3.33	1.17 to 5.49	**0.003**	3.15	1.44 to 4.87	**0.0003**
logACR	222	− 1.04	− 1.88 to − 0.20	**0.02**	− 0.91	− 1.44 to − 0.38	**0.0007**	− 0.87	− 1.32 to − 0.43	**0.0001**
Age	300	− 0.11	− 0.18 to − 0.05	**0.0009**	− 0.10	− 0.13 to − 0.06	**< 0.0001**	− 0.10	− 0.13 to − 0.06	**< 0.0001**
Sex	300	− 2.21	− 4.36 to − 0.06	**0.044**	1.20	− 0.01 to 2.42	**0.05**	1.07	0.00 to 2.15	**0.05**
BMI	284	− 0.10	− 0.26 to 0.05	0.2	0.02	− 0.07 to 0.11	0.7	− 0.01	− 0.08 to 0.09	0.8
Type 2 Diabetes	280	− 1.64	− 3.98 to 0.70	0.17	− 1.97	− 3.31 to − 0.62	**0.004**	− 1.88	− 3.07 to 0.69	**0.002**
HbA1c (%)	228	− 1.36	− 2.32 to − 0.40	**0.005**	− 0.80	− 1.56 to − 0.03	**0.04**	− 0.88	− 1.55 to − 0.20	**0.01**

BP, blood pressure; SBP systolic blood pressure; DBP, diastolic blood pressure; N, number of eyes included in the model; CI, confidence interval; PWV, carotid-femoral pulse wave velocity; eGFR, estimated glomerular filtration rate; ACR, albumin-to-creatinine ratio; BMI, body mass index. Poorly controlled BP defined as mean ambulatory SBP 140 mmHg or DBP 90 mmHg. HbA1c, glycated hemoglobin. Data were analyzed using linear regression models with generalized estimating equation. Bold face indicates statistically significant after multiple testing adjustment.

**Figure 4:**
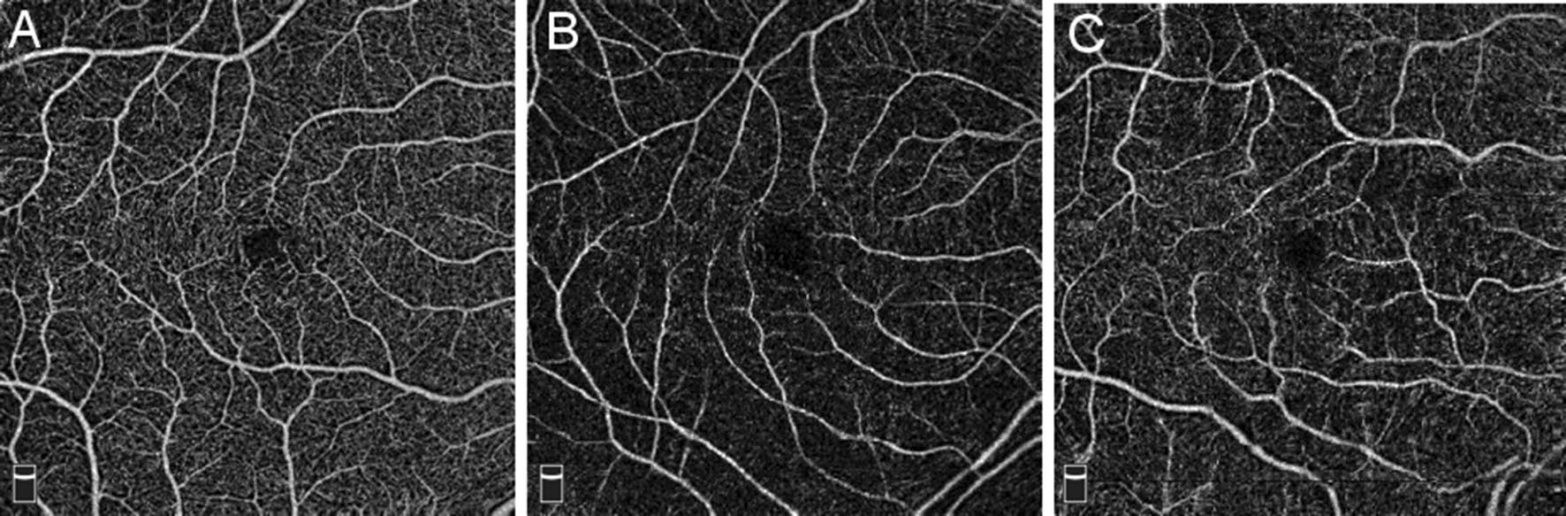
Superficial macular OCT-A image samples demonstrating differences in capillary density. (**a**) High capillary density (foveal density 51.5) in a participant with normal vascular stiffness (PWV 8.8 m/s) and healthy kidney function (eGFR = 90 mL/min/1.73 m^2^). (**b**) Low capillary density (foveal density 21.7) in a participant with high vascular stiffness (PWV 12.2 m/s). (**c**) Low capillary density (foveal density 29.7) in a participant with impaired kidney function (eGFR = 29 mL/min/1.73 m^2^).

Examples of superficial macular OCT-A images are provided in Fig. 4, comparing an image with high capillary density from a participant with normal vascular stiffness and healthy kidney function to an image with low capillary density from a participant with high vascular stiffness and impaired kidney function.

Group comparison for eGFR < 60 (reference eGFR ≥ 60) supported the association with retinal capillary rarefaction (*P* = 0.0003 to *P* = 0.007). Retinal capillary density was not directly associated with ambulatory systolic BP; however, it was associated with diastolic BP for the parafovea and whole image (*P* = 0.01). Retinal capillary rarefaction in the parafovea and whole image was associated with the diagnosis of T2D (*P* = 0.002 and *P* = 0.004 respectively). Retinal capillary rarefaction was associated with higher HbA1c in all 3 zones of interest (*P* = 0.01 to *P* = 0.005). The statistically significant results remained significant after multiple testing adjustment.

The linear associations between retinal capillary rarefaction and measures of HMOD were robust to adjustment for age, sex, SBP, DBP and T2D (see Table 3), with the exception of the foveal density and logACR association (*P* = 0.08). The eGFR group comparisons were also robust to similar adjustment in the parafoveal and whole image zones, but not the foveal zone (*P* = 0.1). The statistically significant results remained significant after multiple testing adjustment.

**Table 3:** Adjusted model results.

	Foveal density			Para foveal density			Whole image density		
	Coefficient	95% CI	*P* value	Coefficient	95% CI	*P* value	Coefficient	95% CI	*P* value
PWV (m/s)	− 0.87	− 1.48 to − 0.27	**0.005**	− 0.40	− 0.79 to − 0.01	**0.04**	− 0.48	− 0.82 to − 0.15	**0.005**
eGFR (mL/min/1.73 m^2^)	0.06	0.00 to 0.11	**0.04**	0.04	0.01 to 0.08	**0.02**	0.04	0.01 to 0.07	**0.006**
eGFR (mL/min/1.73 m^2^) (eGFR > 90 excluded)	0.08	0.02 to 0.15	**0.01**	0.05	0.01 to 0.09	**0.02**	0.05	0.02 to 0.08	**0.002**
eGFR (< 60 mL/min/1.73 m^2^) (reference eGFR ≥ 60)	2.55	− 0.62 to 5.72	0.1	1.99	− 0.01 to 4.00	**0.05**	1.90	0.26 to 3.53	**0.02**
logACR	− 0.76	− 1.61 to 0.10	0.08	− 0.71	− 1.18 to − 0.24	**0.003**	− 0.65	− 1.01 to − 0.28	**0.0005**

Adjusted for age, sex, MAP and type 2 diabetes. MAP, mean arterial blood pressure; CI, confidence interval; PWV, carotid-femoral pulse wave velocity; eGFR, estimated glomerular filtration rate; ACR, albumin-to-creatinine ratio. Data were analyzed using linear regression models with generalized estimating equation. Bold face indicates statistically significant after multiple testing adjustment.

Foveal density was more strongly correlated with HbA1c than T2D (in contrast to the parafovea and whole image density), hence we fitted an additional multivariate model adjusting for age, sex, SBP, DBP and HbA1c instead of diabetes. This did not change the outcomes considerably, as summarized in Table 4.

**Table 4:** Alternative model adjusting for HbA1c.

	Foveal density			Para foveal density			Whole image density		
	Coefficient	95% CI	*P* value	Coefficient	95% CI	*P* value	Coefficient	95% CI	*P* value
PWV (m/s)	− 0.84	− 1.42 to − 0.26	**0.004**	− 0.40	− 0.79 to − 0.01	**0.04**	− 0.48	− 0.82 to − 0.15	**0.005**
eGFR (mL/min/1.73 m^2^)	0.06	0.01 to 0.11	**0.03**	0.04	0.01 to 0.08	**0.02**	0.04	0.01 to 0.07	**0.006**
eGFR (mL/min/1.73 m^2^) (eGFR > 90 excluded)	0.08	0.02 to 0.15	**0.01**	0.05	0.01 to 0.09	**0.02**	0.05	0.02 to 0.08	**0.002**
eGFR (< 60 mL/min/1.73 m^2^) (reference eGFR ≥ 60)	1.00	− 2.06 to 4.07	0.5	1.99	− 0.01 to 4.00	**0.05**	1.90	0.26 to 3.53	**0.02**
logACR	− 0.76	− 1.61 to 0.10	0.08	− 0.71	− 1.18 to − 0.24	**0.003**	− 0.65	− 1.01 to − 0.28	**0.0005**

Alternative adjusted model results for foveal density, replacing the covariate ‘type 2 diabetes” with “HbA1c” (glycated hemoglobin). CI, confidence interval; PWV, carotid-femoral pulse wave velocity; eGFR, estimated glomerular filtration rate; ACR, albumin-to-creatinine ratio. Data were analyzed using linear regression models with generalized estimating equation. Bold face indicates statistically significant after multiple testing adjustment.

These findings indicate that retinal capillary rarefaction in SVP is associated with arterial stiffness as measured by PWV, and impaired kidney function as measured by eGFR and ACR, independent of age, sex, diabetes and best practice BP measures.

## Discussion

The results from our study indicate that microvascular changes in the eye as assessed by retinal capillary density using OCT-A, are clearly associated with other well-established markers of HMOD including arterial stiffness as measured by PWV, and impaired kidney function as assessed both by eGFR and ACR. Importantly, in the context, these associations were independent of age, sex, diabetes and ambulatory BP measures. Retinal capillary rarefaction may therefore represent a useful and simple test to assess the integrated burden of hypertension on the microvasculature irrespective of current BP levels and therefore add substantially to risk stratification of hypertensive patients. Furthermore, future research will have to investigate whether longitudinal assessment and the corresponding changes in the retina may serve as an indicator of adequate/inadequate antihypertensive treatment.

A recent similar small study applying OCT-A in non-diabetic hypertensive patients of Asian ethnicity was reported by Chua et al.^25^. The Chua study found an association between BP and foveal density at the deep vascular plexus (DVP). It did not investigate PWV but found an association between foveal density and eGFR, similar to our result but only statistically significant for the DVP, not SVP. Findings from our study in a larger cohort of patients suggests that the association between eGFR and foveal density holds also for the SVP (and parafoveal and full image zones) and provides the first evidence that PWV is associated with retinal capillary density. There was agreement between the studies on the lack of association at the SVP layer between foveal density and 24 h ambulatory BP. However, in extending the zones of interest we found associations between retinal capillary density and DBP in the parafovea and whole image. We provide evidence therefore that hypertension may similarly affect both SVP and DVP layers, akin to other systemic disorders such as diabetes^24^.

Retinal capillary rarefaction has previously been reported in diabetes^28^, along with retinal microvascular changes in pre-diabetes^29^. Our results provide further evidence of retinal capillary rarefaction in diabetes (in parafovea and whole image) and indicate a linear relationship between HbA1c and capillary density in all 3 zones of interest.

A previous study found increased PWV was associated with wider retinal venular caliber in the Atherosclerosis Risk in Communities Study^30^. Current guidelines by the European Society of Hypertension state that a measured PWV larger than 10 m/s can be considered an independent marker of HMOD^8^. Interestingly, our study suggests that the fine retinal capillaries may also provide some information about the health of the large arteries, such as the aorta, as assessed by PVW. However, this is based on correlation analysis only and will require further substantiation. Nevertheless, the association of retinal capillary density with markers of both micro- and macro-vascular damage indicates that this measure may represent an attractive approach to better understand and quantify the overall/integrated burden of elevated BP over prolonged periods of time on susceptible vascular structures and provide superior assessment of HMOD with potential therapeutic implications.

The capillaries are more representative of the entire microvascular network and may provide the earliest markers of ischemia/hypoxia, before arteriolar and venular changes occur. Early detection of hypertensive damage is important to both CV risk stratification and to prevent further retinal degeneration. Indeed, recent evidence suggests that retinal neurodegeneration is already evident in individuals with only mildly elevated BP^23^.

Retinal capillary rarefaction was independently associated with impaired kidney function (reduced eGFR and logACR). The retinal and renal circulations exhibit shared characteristics in terms of anatomy and physiology^31^, hence it is possible that retinal capillary rarefaction reflects similar changes in the kidney in hypertension. The results are consistent with previously published associations between retinal arteriolar narrowing and kidney function^32,33^ or kidney disease^19,20,34^. Future research will be needed to determine if retinal capillary rarefaction, compared to arteriolar narrowing, can provide an earlier or more accurate marker of hypertensive damage including kidney dysfunction.

As OCT-A is non-invasive, accessible and relatively low cost, it could be a very useful and simple addition to the routine diagnostic workup of hypertensive patients in the future. Current guidelines recommend screening for HMOD in multiple organs^8^, or multiple markers for a single organ in the case of the kidney (eGFR and ACR), because CV risk increases for each extra sign of HMOD^35^. Future longitudinal research is required to establish whether retinal OCT-A markers improve CV risk stratification over existing markers, or provide an easier method compared to less accessible or more costly tests.

Associations between retinal capillary density and measures of HMOD were substantially stronger than those with best practice BP measures, i.e. ambulatory BP, in this treated hypertension cohort. This was true for univariate and adjusted models. Indeed, for the fovea region there was no association between retinal capillary density and BP. Hence, we hypothesize that the correlations between retinal density and measures of HMOD reflect cumulative damage due to previous BP burden.

Even ABPM, while superior in predicting CV outcomes, is only a snapshot of the BP profile at a specific point in time. It does not necessarily inform about longer term BP control and prevention of further progression or even regression of HMOD. OCT-A imaging could potentially fill this gap and provide an integrative marker for previous BP burden, similar to the use of HbA1c in assessing control of glucose metabolism over the previous ~ 3 months in patients with diabetes. An integrated measure of the BP burden on both the micro-and macro-vasculature may be most useful to improve individual management of hypertension and potentially to monitor treatment effects. This, however, will have to be addressed in appropriately designed longitudinal studies.

The OCT-A measures of retinal capillary density are based on optical changes due to blood flow; if blood flow drops below the detection threshold, the capillary will not be identified and the measured density will be reduced^36^. A different retinal imaging modality, laser doppler flowmetry, has also been used to demonstrate reduced retinal capillary density in hypertensive patients^37^, along with increased retinal vascular resistance^38^. Hence it is possible that atherosclerotic processes that reduce blood flow are the mechanism by which retinal capillary rarefaction occurs in hypertension. Of note, foveal vascular density is anatomically influenced by the foveal avascular zone which can lead to increased variability of density estimates. Therefore, results of the para-foveal density and whole image density may be more robust.

The recent development of OCT-A technology, permitting fast, non-invasive assessment of the retinal capillaries and blood flow, is now being utilized extensively for clinical research in many disorders including ocular disease. Our results add to the evidence of retinal capillary changes due to hypertension, highlighting that clinical researchers exploring OCT-A in ocular diseases should either exclude or adjust for systemic disorders such as hypertension and diabetes.

The strengths of this study include a well phenotyped cohort including ambulatory BP monitoring, PWV and kidney function (eGFR and ACR). The results from this cross-sectional study warrant further investigation with longitudinal studies. One limitation of the study was the inclusion of patients on different medications and different stages of hypertension, however this reflects a real-world hypertensive cohort. Additionally, the duration of hypertension was not exactly known, and the associations do not prove causality.

We have demonstrated a relationship between retinal capillary density and both macro- (PWV) and micro-vascular (eGFR and ACR) parameters in adults with treated arterial hypertension. Assessment of the microcirculation is crucial to the understanding of the effects of systemic diseases such as hypertension and diabetes^39^. As with other target organ damage in hypertension, recognition of the effects on the retinal capillaries could allow clinicians to better manage hypertensive patients. OCT-A is a practical, non-invasive tool for investigation of the retinal capillaries which may mirror other HMOD such as microvascular rarefaction in the kidney and stiffening of large arteries. As such, assessment of retinal capillary density may have clinically relevant advantages in that it may represent a superior marker of overall vascular damage (both micro- and macrovascular) and may serve as a stand-alone and powerful integrated measure of HMOD irrespective of the current BP level.

## Materials and methods

### Study design and participants

We conducted a prospective cross-sectional study including 155 participants with arterial hypertension from the Dobney Hypertension Centre at the Royal Perth Hospital, Perth, Australia. Patients aged 18 years or older underwent OCT-A imaging between February 2016 to October 2019. All images were evaluated by an eye specialist, participants with retinal disease (glaucoma, age-related macular degeneration, diabetic retinopathy) or previous retinal surgery (n = 5) were excluded, leaving *n* = 150 participants for the current analysis. The study was approved by the East Metropolitan Health Service Ethics and Governance Committees (EC00270, REG Number: RGS1040). The research followed the tenets of the Declaration of Helsinki for research involving human subjects. Informed consent was obtained from all subjects.

### Clinical workup

Patients were referred to the Dobney Hypertension Centre for management of hypertension predominantly from primary care physicians. Clinical diagnosis for hypertension was based on current European guidelines^40,41^. A thorough medical history was obtained from all patients and a routine physical examination was performed. Secondary causes of hypertension were investigated where clinically indicated. Patients with identified secondary hypertension were not included in this analysis. Office BP measurements were obtained according to unattended automated office BP principles and the average of three measurements recorded.

All participants were fitted with an ambulatory BP monitor by a trained research nurse. The devices (Spacelabs 90207, Spacelabs Healthcare, Snoqualmie, Washington, United States) were programmed to take 4 measurements per hour during the day (6:00 – 22:00 h) and 2 per hour during the night (22:00–6:00 h). Arm circumferences were measured and appropriate cuffs were chosen according to Spacelabs product specifications and the first reading was performed in the presence of research staff.

Pulse wave velocity (PWV) measurements were carried out with SphygmoCor® XCEL system (AtCor Medical Pty Ltd, Australia). Standard procedures of the manufacturer were followed to obtain the parameters. Participants were asked to attend fasted on the morning of the assessment and to abstain from smoking, caffeine and alcohol consumption 12 h before the assessment and not to take their usual BP medication. Participants were instructed to take their regular BP medication after the assessment of PWV and retinal imaging, which was usually carried out in the morning (before 12:00 h). The PWV was performed after a 5-min rest period in supine position. Parallel assessment of pulse in thigh with specialized cuffs and a mechanical sensor held against the neck of the participants allowed calculation of PWV^42^. The distance in between the sites where the pulse was recorded was measured by trained study nurses or doctors before the measurement. The capturing time for PWV assessment was set to 10 s with a PWV distance and subtraction method. The PWV assessments was performed twice and their average used for further analysis. For analysis purposes, an additional parameter “BP control status” was defined as poorly controlled if mean ambulatory systolic blood pressure (SBP) > 140 mmHg, or diastolic blood pressure (DBP) > 90 mmHg.

### Pathology

Blood samples and mid-stream urine were obtained from participants within 4–6 weeks prior to retinal imaging. If participants had already obtained the required pathology results within 3 months prior to retinal imaging these results were used for analysis.

Pathology tests included serum creatinine, spot urine albumin and calculation of eGFR (measured with the CKD-EPI algorithm^43^ in mL/min/1.73 m^2^) and ACR (Albumin/Creatinine ratio in mg/mmol creatinine) fasting glucose and HbA1c_._

### Retinal examination procedures

Participants completed a questionnaire regarding their ocular history, including glaucoma, macular degeneration and intraocular surgery. Retinal imaging was performed using the Optovue AngioVue® spectral-domain optical coherence tomography-angiography (OCT-A) device (Optovue, Inc., Fremont, CA, USA). Non-invasive OCT-A technology uses laser reflectance of the surface of flowing red blood cells to accurately depict vessels in the retina, including fine capillaries, without the traditional need for intravascular dyes^44^. As with traditional OCT, cross-sectional scans are combined to provide 3-dimensional image data, however with OCT-A, changes in signal over time are used to detect blood flow, permitting quantitative measurement of flow area and vessel density. The OCT-A algorithms produce 2-dimensional images (en face angiograms or capillary density maps) segmented into four retinal depths: the superficial retinal plexus (SVP), the deep retinal plexus (DVP), the outer retina and the choriocapillaris (Fig. 1). The retinal capillaries exist primarily in the SVP and DVP layers. The AngioVue system uses split-spectrum amplitude-decorrelation angiography algorithm, which minimizes motion noise. A high level of reproducibility for retinal capillary density measurements using OCT-A has already been published^45^.

The OCT-A device was used to collect macular scans with a 6 mm × 6 mm field of view. AngioVue software (version 2016.1.0.26) automatically provided segmentation of the SVP and calculated the percentage image area occupied by blood vessels with flowing erythrocytes in the selected region; the superficial retinal capillary density.

A trained grader blinded to the participant characteristics reviewed the quality of all OCT-A scans. Poor quality scans were excluded from the analysis if they exhibited segmentation failure or image artifacts due to optical or residual motion.

For this study, capillary density at SVP was measured in macula region, in a 6 mm square region centered on the fovea. The SVP includes the vasculature between the internal limiting membrane and 10 µm above the inner plexiform layer. Capillary density was calculated for 3 regions of interest, each centered on the fovea (see Fig. 2); the whole image, the fovea (a circular region 1.5 mm in diameter centered on the fovea) and parafovea (an annulus extending from the outside of the fovea region out to a diameter of 2.5 mm).

### Statistical analyses

Baseline demographic and clinical characteristics were reported as absolute numbers and relative proportions in percent for categorical data and mean and standard deviation (SD) for continuous data.

To test for associations between retinal capillary density and HMOD parameters, generalized linear modelling (GLM) was utilized, with retinal capillary density as the dependent variable. We found a moderate positive correlation between right and left eye data on retinal density (Pearson correlation coefficient, r = 0.68; *P* < 0.0001). Hence, we utilised the inter-eye variability by considering each eye as the unit of analysis, providing greater statistical power. The GLM analyses were modified according to the generalized estimating equation (GEE) approach^46^. The GEE provides increased precision of estimation results while accounting for the correlation between fellow eyes. Benjamini and Hochberg^47^ multiple testing adjustment (false discovery rate set to 0.1) was applied to the retinal capillary density results (foveal, parafoveal and whole image).

Where unadjusted models were significant, multivariate models were fitted including adjustment for age, sex, mean arterial pressure (MAP) and T2D; only covariates with *P* < 0.1 were retained in the models. An additional model was provided for foveal density, replacing the covariate T2D with HbA1c, as HbA1c displayed stronger association with foveal density than T2D. HbA1c can be used to identify pre-diabetes and diabetes^27^.

Data for eGFR were not numerically provided above 90 mL/min/1.73 m^2^, as values above 90 mL/min/1.73 m^2^ are less accurate. To avoid introducing bias in the linear regressions, participants with normal eGFR were excluded from this particular analysis. Additionally, parameters of renal damage were also used as categorical, independent variables for linear regression models with main retinal vascular outcome markers as dependent variables to test for group differences. Logarithmically transformed ACR was used for linear regressions.

Statistical significance was defined as p < 0.05.

All statistical analyses were conducted in the R statistical environment^48^.

## Data Availability

The data that support the findings of this study are subject to restrictions and are not being made publicly available. Data are however available from the authors upon reasonable request and with permission of the hospital and ethics committee.
